# Cold Atmospheric Pressure Plasma (CAP) as a New Tool for the Management of Vulva Cancer and Vulvar Premalignant Lesions in Gynaecological Oncology

**DOI:** 10.3390/ijms21217988

**Published:** 2020-10-27

**Authors:** Pavol Zubor, Yun Wang, Alena Liskova, Marek Samec, Lenka Koklesova, Zuzana Dankova, Anne Dørum, Karol Kajo, Dana Dvorska, Vincent Lucansky, Bibiana Malicherova, Ivana Kasubova, Jan Bujnak, Milos Mlyncek, Carlos Alberto Dussan, Peter Kubatka, Dietrich Büsselberg, Olga Golubnitschaja

**Affiliations:** 1 Department of Gynaecological Oncology, The Norwegian Radium Hospital, Oslo University Hospital, 0379 Oslo, Norway; yunwang@ous-hf.no (Y.W.); anndoe@ous-hf.no (A.D.); 2 OBGY Health & Care, Ltd., 010 01 Zilina, Slovakia; 3 Department of Medical Biology, Jessenius Faculty of Medicine, Comenius University in Bratislava, 03601 Martin, Slovakia; alenka.liskova@gmail.com (A.L.); marek.samec@gmail.com (M.S.); koklesova5@uniba.sk (L.K.); peter.kubatka@uniba.sk (P.K.); 4 Biomedical Centre Martin, Jessenius Faculty of Medicine, Comenius University in Bratislava, 03601 Martin, Slovakia; zuzana.dankova@uniba.sk (Z.D.); dana.dvorska@uniba.sk (D.D.); vincent.lucansky@uniba.sk (V.L.); bibiana.malicherova@uniba.sk (B.M.); ivana.kasubova@uniba.sk (I.K.); 5 Department of Pathology, St. Elizabeth Cancer Institute Hospital, 81250 Bratislava, Slovakia; kkajo@ousa.sk; 6 Department of Obstetrics and Gynaecology, Kukuras Michalovce Hospital, 07101 Michalovce, Slovakia; janbujnak@hotmail.com; 7 Department of Obstetrics and Gynaecology, Faculty Hospital Nitra, Constantine the Philosopher University, 949 01 Nitra, Slovakia; mlyncekmilos@hotmail.com; 8 Department of Surgery, Orthopaedics and Oncology, University Hospital Linköping, 581 85 Linköping, Sweden; cadussanl@gmail.com; 9 Department of Physiology and Biophysics, Weill Cornell Medicine-Qatar, Education City, Qatar Foundation, P.O. Box 24144 Doha, Qatar; dib2015@qatar-med.cornell.edu; 10 Predictive, Preventive Personalised (3P) Medicine, Department of Radiation Oncology, Rheinische Friedrich-Wilhelms-Universität Bonn, 53105 Bonn, Germany; Olga.Golubnitschaja@ukbonn.de

**Keywords:** cold atmospheric plasma, gynaecological oncology, vulva cancer, risk factors, plasma tissue interaction, premalignant lesions, cancer development, patient stratification, individualised profiling, predictive preventive personalised medicine (PPPM/3PM), treatment

## Abstract

Vulvar cancer (VC) is a specific form of malignancy accounting for 5–6% of all gynaecologic malignancies. Although VC occurs most commonly in women after 60 years of age, disease incidence has risen progressively in premenopausal women in recent decades. VC demonstrates particular features requiring well-adapted therapeutic approaches to avoid potential treatment-related complications. Significant improvements in disease-free survival and overall survival rates for patients diagnosed with post-stage I disease have been achieved by implementing a combination therapy consisting of radical surgical resection, systemic chemotherapy and/or radiotherapy. Achieving local control remains challenging. However, mostly due to specific anatomical conditions, the need for comprehensive surgical reconstruction and frequent post-operative healing complications. Novel therapeutic tools better adapted to VC particularities are essential for improving individual outcomes. To this end, cold atmospheric plasma (CAP) treatment is a promising option for VC, and is particularly appropriate for the local treatment of dysplastic lesions, early intraepithelial cancer, and invasive tumours. In addition, CAP also helps reduce inflammatory complications and improve wound healing. The application of CAP may realise either directly or indirectly utilising nanoparticle technologies. CAP has demonstrated remarkable treatment benefits for several malignant conditions, and has created new medical fields, such as “plasma medicine” and “plasma oncology”. This article highlights the benefits of CAP for the treatment of VC, VC pre-stages, and postsurgical wound complications. There has not yet been a published report of CAP on vulvar cancer cells, and so this review summarises the progress made in gynaecological oncology and in other cancers, and promotes an important, understudied area for future research. The paradigm shift from reactive to predictive, preventive and personalised medical approaches in overall VC management is also considered.

## 1. Introduction

Cold atmospheric plasma (CAP) is a highly reactive ionised physical state containing a mixture of physical and biologically active agents. The basic components are the variety of reactive oxygen and nitrogen species formed on reaction with molecules (oxygen, nitrogen, and water) present in the ambient air [1]. Plasma-derived reactive species are free radicals, including oxygen forms (ozone O_3_, superoxide anion O_2_^−^), hydroxyl radical (OH), hydrogen peroxide (H_2_O_2_), nitrogen dioxide radical (NO_2_), nitric oxide (NO), peroxynitrite (ONOO^−^), organic radicals, electrons, energetic ions, and charged particles [2,3,4,5,6,7].

Study of their interaction with biological cell or tissue components revealed that biological plasma effects are mediated via reactive oxygen (ROS) and nitrogen species (RNS) which affect cellular redox-regulated processes [8,9], initiating many cellular responses with selectively-targeted anti-tumour effects (e.g., inhibition of cell adhesion, selective apoptosis, necrosis or the inhibition of cell proliferation by disrupting the S-phase of cell replication in tumour cells, suppression of metastatic cell migration, induction of membrane permeation or inducing lethal DNA damage) [10]. 

The mechanisms underlying this selective cancer cells killing are explained as follows: cancer cells are characterised by a more active metabolic status, resulting in higher basal ROS and RNS levels, making these cells more susceptible to the oxidative stress added by CAP, and especially when cancer cells express high DNA replication activity and there is a high percentage of cells in the S-phase [11,12,13]. This CAP effect on cancer cells can be further augmented by synergic combination with PAM-nanoparticles (plasma activated medium) [14]. The second obvious result is the significant technical progress in tools allowing CAP application in medicine [15]. All these data show that CAP is beginning to be adopted as a new tool in biomedicine.

CAP operates at body temperature, making it feasible for a variety of medical applications, such as chronic wound treatment; skin disinfection [16,17,18]; tissue regeneration in chronic leg ulcers [19]; dentistry [20]; in dermatology for the treatment of tumours, actinic keratosis, scars, ichthyosis, psoriasis, atopic eczema, as well as for alleviation of pain and itch [21,22,23]; and in haematology for blood coagulation [24,25]; in ophthalmology (human corneas) [26]. Recently there has been increased interest in clinical applications in anticancer therapy as a novel promising treatment [27], leading to a new field of medicine called “plasma oncology or plasma medicine” [8,28,29]. 

The evidence from translational and clinical studies of CAP effects on cancer cells or solid tumours has allowed the extensive use of CAP in the clinical management of cancer patients through both intraoperative and postoperative application for local tumour control. CAP applications in oncology have shown remarkable anticancer effects in vitro cell-lines, including, for example, melanoma [30], cutaneous squamous carcinoma [31], pancreatic [32], liver [33], gastric [34], colon [35], prostate or urinary bladder [36,37], breast [38,39,40,41,42,43,44], head and neck cancer [45], osteosarcoma [46,47], glioblastoma [48], lymphoma [49], acute myeloid leukaemia [50], multiple myeloma [51], human fibrosarcoma [52], or lung cancer [53], as well as in vivo solid tumour types in animal (mice) models, e.g., colon [54], breast [55,56], prostate cancer [57], cholangiocarcinoma [58], schwannoma [59], glioblastoma [60], or melanoma [61]. A limited number of studies have been published in oncogynaecology, however, mostly restricted to in vitro cell lines, e.g., cervical [12,62,63,64,65,66,67,68,69], endometrial [70,71,72], or ovarian [11,73,74,75,76,77,78].

Vulva cancer and vulvar premalignant lesions (VIN) are suitable for the broad clinical application of CAP in an anticancer approach using therapeutic strategies for the following specific reasons:

(a) Vulva cancer is technically easy to approach using CAP.
(b) The effect of radioresistance in subtypes of this malignancy is becoming a clinical problem.
(c) VIN lesions are commonly treated/managed with local drugs or by applying tracer, which may be suitable for large PAM (plasma-activated medium) treatment.
(d) Recovery from postoperative vulva surgical site wounds is often prolonged, requires special nursing, and is often combined (in 30–75%) with microbial infections [79,80] in need of antibiotics, whereas the antibacterial effect of CAP may facilitate the healing process.
(e) Anatomical circumstances usually restrict re-excisions after primary surgery, which is often combined with advanced plastic flaps (e.g., in the case of “worrisome” surgical margins).
(f) The most common type of vulvar cancer is skin squamous carcinoma (70%) [81,82], followed by melanoma (10%) [83,84] and extramammary Paget disease (1–2%) [85,86], for which CAP has already been clinically validated on both cell lines and human tumours.

The current cancer treatment is focused on the complete surgical eradication of cancer cells and minimum non-malignant tissue. It is difficult to obtain satisfactory free surgical margins in vulvar cancer due to its anatomical specificity and in some cases the close location to the urethra and anus. Despite the intentions of radical excision, moreover, there may be a risk of microscopic tumour residue or local spreading beyond surgical margins, and adjuvant treatment with re-excision or radiation/chemoradiation therapy may be required. Importantly, the majority of these patients are elderly, with comorbidities and reduced wound healing. Conversely, patients with vulva cancer precursors are often young, and the various repeated treatments throughout their lives, including skinning surgery or laser treatment, are associated with a risk of developing dyspareunia due to fibrosis, fissures, and loss of normal anatomy. There is thus a need for more specific treatment modalities for vulva cancer.

The PubMed database was searched up until 15th August 2020 to determine the current knowledge of CAP in oncogynaecology, its technological level, and the biology of tissue interactions, using the search terms “cold atmospheric plasma” and “cancer” (in vitro, in vivo, clinical trials, case reports), resulting in 265 matched articles. Relevant papers included in this systematic review were obtained from the English-language literature, mostly dating from 2015-2020. Specific databases related to plasma physics were also reviewed (American Institute of Physics (AIP)**,** IOPscience, IEEE Xplore), including journals focusing on plasma in Scopus, Elsevier, and the Wiley Online Library.

In-depth analysis of the articles showed that plasma studies were mostly conducted in vitro and concerned direct plasma treatments, followed by PAM. In vivo studies were dominantly performed on mice models. Only sporadic clinical studies have been recorded, mostly in dermatology or head and neck malignancies. The data related to gynaecological cancer were scarce. This review thus offers an overview of CAP-related plasma medicine for female malignancies, and especially vulvar cancer.

The review aims to summarise the potential of CAP for the clinical treatment of vulvar cancer and VIN, as it has not been reported previously, apart from sporadic studies on cell lines confirming the anticancer effect of CAP on cellular proliferation, apoptosis, necrosis, or migration. Our study also provides a comprehensive overview of CAP biology, its interaction with the tissues, the origin of biological processes that are crucial steps in carcinogenesis and surgical wound healing, as well as insight into the modern approaches based on CAP for future medicine. The clinical importance of such reviews is now emerging, and plasma medical devices are widely used in current practice, such as the plasma jet kINPen or InvivoPen [87,88]. The benefits of CAP in clinical application are increasing, most recently in immunotherapy [89,90], and in the combination of CAP and nanoparticles [27,40,91]. CAP thus seems to be an auspicious tool for the development of a new cancer treatment strategy in vulva oncology. Non-thermally operated plasma sources could also be a suitable alternative for the treatment of precancerous and cancerous lesions in gynaecological oncology especially, due to small size and high flexibility of the application probes.

## 2. Epidemiology and the Prevalence of Vulvar Cancer

Vulvar cancer is a rare disease, accounting for some 5–6% of all gynaecological cancers, but is the fifth most common cancer type after uterine corpus, ovarian, cervical, and vaginal cancer, with breast cancer as the most common malignancy in women. Almost 60% of patients are diagnosed at an early stage, without evidence of local lymph node metastasis and infiltration of the surrounding tissue [92,93]. This malignancy often affects older women, between 60–75 years, and around 90% of all vulvar cancers are vulvar squamous cell carcinomas (VSCC) [94,95,96,97,98]. The incidence of vulvar cancers ranges from 0.6–1.0 cases per 100,000 women and has increased profoundly since the 1970s [99,100]. This trend has been observed not only for postmenopausal women, but also in younger women (almost doubled in 30–49 year age group) because of the increase in HPV-mediated disease [101,102], accounting for 34–40% of vulvar cancers [103,104,105], and immunocompromising conditions in patients, such as renal transplant recipients [106]. 

A national Norwegian study reported that prevalence has increased in recent decades (>2.5 times), especially among women under 60 (by 150% in the 0–39 year age group, 175% in the 40–49 year age group and 68% in the 50–59 year age group). One factor discussed was altered sexual activity at young ages without the use of condoms. Although the incidence of VSCC has been increasing for decades in most Western countries, there has conversely been a decreasing trend in some southern European states [107].

More precise knowledge of tumour biology and improvements in therapeutic approaches has resulted in less aggressive surgical treatments in clinical practice, with improved survival [94]. The most important prognostic indicator for survival in women with vulvar cancer is inguinofemoral nodal involvement [108] and, deep multivariate analysis of prognostic factors in primary VSCC also indicates newly assessed perineural invasion. This last parameter was determined as the relevant independent prognostic factor for aggressive behaviour and an unfavourable course in VSCC that should be considered in adjuvant treatment planning [109]. The five-year overall survival rate for localised early-stage vulvar cancer (Stage I/II) varies from 86–90%, to 52.6–60% for locally advanced forms or with locoregional groin lymph node metastatic extension (stages III/IVA), decreasing to 20-22.7% for cases with distant metastases (stage IVB) [110,111]. The age-standardised mortality rate for vulvar cancer in Europe is stated as 0.7/100,000 women [99], and worldwide is 0.3/100,000 [100]. The number of women with high-grade VIN tripled during the last decade (five per 100,000 women), mostly in the HPV-related type [112]. In women ≤ 50 years old, the incidence of high-grade VIN increased by four, and of invasive vulvar cancer by 1.6 [113,114].

## 3. Aetiopathology, Clinical Aspects and Current Treatment of Vulvar Cancer and Its Premalignant Lesions 

### 3.1. Precursors and Classification of the Disease

VSCC initially develops from squamous precursor lesions of the vulva, which are referred to as vulvar intraepithelial neoplasias (VIN), which were initially graded as VIN1, VIN2 and VIN3; the additional VIN3 differentiated type was also introduced recently [115]. VIN1 was removed in recognition of the aetiological and prognostic differences from histopathological, molecular, and clinical studies, due to its negligible risk for cancer progression. A two-tier classification scheme was proposed: (1) uVIN (usual VIN), including lesions previously classified as VIN2 and VIN3, and (2) dVIN (differentiated or simply VIN) [82]. 

The precursor lesions of VSCC associated with HPV-infection are currently classified as: (1) low-grade squamous intraepithelial lesion (SIL) of the vulva or vulvar LSIL, encompassing flat condyloma or human papillomavirus effect, and (2) high-grade SIL or vulvar HSIL (which was termed uVIN). The vulvar intraepithelial neoplasia differentiated type (dVIN) is the HPV-unrelated precursor lesion of VSCC [116]. Only HSIL/uVIN and dVIN are considered premalignant lesions for vulvar cancer, with a significantly increased incidence in recent decades [114]. 

These two different pathways with their own precursor lesions are those that have been identified so far in the development of VSCC, based on detailed histological, immunohistochemical, and genetic abnormalities providing genetic evidence for a clonal relationship between VSCC and its precursors. The first pathway is associated with lichen sclerosis (LS) or other chronic vulvar dermatoses [117,118,119,120], and dVIN (HPV-independent VIN) [121], correlated with a higher invasive malignancy risk, mutations of p53-p16(INK4a) and the retinoblastoma tumour suppressor gene involved in the process of malignant transformation [122]. The dVIN is the precursor lesion of keratinising SCC, which is the most common subtype of invasive SCC, accounting for 63–86% of all cases of VSCC [118]. The second pathway is caused by a persistent human papillomavirus (HPV) infection (mostly HPV type 16, 33, and 18), with HSIL/uVIN as the associated precursor of warty and basaloid invasive SCC [116], but with better prognosis, longer disease-free survival [123] and better response to radiotherapy [124] than HPV-negative ones, and this is the same for the invasive form of vulvar cancer [103]. 

Both precursors, HSIL/uVIN and dVIN, show different risks of progression from that of invasive VSCC. The rate of progression from HSIL/uVIN to VSCC has been reported as less than 5%, but dVIN progresses to invasive VSCC in up to 35% of cases [125]. 

Traditionally, histology and immunohistochemistry (IHC) have been the basis of the diagnosis and classification of VIN. HSIL/uVIN shows conspicuous histological atypia and positivity on p16-IHC, whereas dVIN shows less obvious histological atypia, and overexpression or a null-pattern on p53-IHC. Other diagnostic immunohistochemical markers have also been evaluated for both types of VIN. The molecular characterisation of VIN has been attempted in a few recent studies, and novel genotypic subtypes of HPV-independent VSCC and VIN have been identified [98].

### 3.2. Current Treatment of the Disease

As the incidence of premalignant vulvar lesions has increased in recent decades, especially in younger women, is it the knowledge of aetiopathology and risk factors that determines its management [114]. The purpose of treatment for vulvar precursor lesions is to relieve symptoms, prevent cancer progression, and preserve anatomy and organ function [126]. The currently preferred treatment modality for HSIL/uVIN or dVIN is surgical excision, or skinning vulvectomy [127]. Recurrence is not uncommon after treatment, however. One study reported a recurrence rate of about 30% and that around 9–18% of patients with high-grade VIN will progress to cancer [128]. The laser vaporisation of small lesions [129] or medical treatment with Imiquimod (Aldara^®^) are alternative treatments, and the complete response rates after Imiquimod treatment ranged from 5% to 88% [130]. 

Surgical treatment is a preferred therapeutic approach in the early stages of vulvar cancer. The standard procedure entails radical local excision of the primary tumour and evaluation of groin lymph node status, either by an elective inguinofemoral lymphadenectomy or sentinel node-dissection, depending on tumour size, focality or the presence of suspected metastatic groin lymph nodes [131]. The adequate clearance of groin lymph nodes is important as recurrence occurs early in the groin, and has repeatedly been reported as fatal, with a median OSR of only 6–10 months [132,133]. Recurrent disease confined to the vulva can be treated with surgical resection only, with cure rates of 20–79%. Here, pelvic exenteration is a therapeutic option with acceptable complication rates for patients with large local recurrences, for whom other treatments are not an option [134]. However, the procedure is associated with a high overall mortality rate. The strict selection of patients is necessary to reach satisfactory surgical and oncologic outcomes. 

As the surgical treatment of VSCC is associated with significant morbidity and high recurrence rates, which are related to the limited ability to distinguish (pre)malignant from healthy tissue, there is a need for new tools for the real-time detection of occult tumour lesions and the localisation of cancer margins in patients with VSCC. Several tumour-specific imaging techniques have thus been developed to recognise malignant tissue by targeting tumour markers [135], and new technologies such as CAP are considered for the elimination of micrometastases. 

An adjuvant radiotherapy should start as soon as possible after surgery when invasive disease extends to the pathological excision margins of the primary tumour, and further surgical excision is not possible, or for cases with more than 1 metastatic lymph node and/or presence of extracapsular lymph node involvement [136,137]. Despite the radical treatment, up to 12–39% of VSCC across all patients (30% local-regional, 18% distant) experience recurrence [81,138,139]. Routine surveillance is recommended following primary treatment. Most recurrences occur within the first two years after treatment: 32.7% of patients with node-positive cancer and 5.1% among women with negative nodes [140]. Patients with nodal metastatic disease recur at the groin at 10.5 months on average [141].

Advanced stage patients should be evaluated in a multidisciplinary setting to determine the optimal choice and order of treatment modalities. Neoadjuvant chemoradiation should be considered in order to avoid exenterative surgery. Definitive chemo-radiation with weekly cisplatin is the treatment of choice in patients with unresectable disease [136,142,143]. The best treatment option for patients with advanced cancer is combined treatment with surgery and radiotherapy ± chemotherapy. Radiotherapy with a dose of ≥54.0 Gy should be considered to achieve better local control if adverse factors are present [144,145]. The GOG 205 trial demonstrated complete clinical response in 78% patients with T3/T4 tumours following chemoradiation [142]. Primary chemoradiation has become the initial treatment choice for locally advanced disease, followed by resection of residual tumour. The management of patients with extrapelvic metastatic disease focuses on palliative care and the improvement of quality of life by chemoradiation and pain-control with supportive care approaches [146]. 

Outside current practice, the importance of novel therapeutic approaches for local disease control is emerging, as data from the AGO CaRE-1 study, with an exceptionally long follow-up of 80 months, confirmed that the pathologic tumour-free margin distance did not affect the risk of local recurrence (12.6% in patients with margins <8 mm and 10.2% in cases with a margin at least 8 mm). No differences in local recurrences were found between patients who did or did not receive adjuvant radiotherapy [147,148,149]. Furthermore, any aim to achieve better local margins control can easily result in mutilation, especially when the primary tumour is located close to the clitoris, as it is in up to 25–37% [102]. This aim could be guaranteed by a peritumoural injection of indocyanine green for the intraoperative identification of surgical margins [150] and CAP application for the selective killing of eventual site micrometastases as a novel tool for a surgeon. This data strengthens the recommendation for a more intense, long-term follow-up for VSCC patients with a history of LS or dVIN [133] and supports the proof of concept for starting studies with CAP for better VSCC control.

## 4. Current Knowledge of In Vitro Cell Lines and Further Potential for Clinical Application of CAP Oncogynaecology

The application of plasma in cancer treatment is currently a highly topical area of research in its many types. New and significant findings have been demonstrated, most of all in the field of skin, head, and neck cancer, as demonstrated in several studies [30,151]. The first clinical study of the local application of CAP was performed by Metelmann et al. (2015) [152] in 12 patients with advanced head and neck cancer and infected ulcerations, followed by palliative treatment. Here, CAP was applied using a plasma jet, kINPen(^®^) MED (neoplas tools GmbH, Greifswald, Germany; 1min/cm^2^, 3 times/week, 1–9 cycles), with very promising results, showing an increased number of apoptotic cells in tissue areas previously treated with CAP compared to untreated areas. In the CAP group the clinical tumour surface response was expressed as a flat area with vascular stimulation or a contraction of tumour ulceration rims, and no patients showed signs of enhanced or stimulated tumour growth. CAP did reduce the bacterial contamination of cancer ulcerations, and eased local cancer pain felt by patients. Surgeons indicated that CAP application by plasma jet was easy to handle and extremely precise [152,153]. This started further clinical oriented studies. Schuster et al. (2016) [153] applied CAP with 21 patients with advanced squamous cell carcinoma of the head and neck, reporting increased proportions of apoptotic cells in CAP-treated tissue compared to non-treated ones; and Canady (2017) [154] used plasma as a tool for surgery to enable the complete removal of gastrointestinal tumours in Stage IV patients, and minimise the incidence of recurrence.

At the same time, the potential of CAP in the treatment of gynaecological oncologic diseases can be illustrated by the example of current studies evaluating, for example, breast [55] or ovarian cancer [73]. There have not been any large clinical studies on CAP in gynaecological malignancies, however, although VSCC or cervical lesions are suitable for its use at large scale [69]. Its clinical benefits for local solid tumour management are also supported by the ability of different plasma sources to penetrate solid biological tissues both in vivo and in vitro [155,156]. These studies showed penetration of reactive species generated in plasma (e.g., hydrogen peroxide) deep into the tissue, allowing to study plasma effect on dirty, oily, bloody, and morphologically complex surface (e.g., features present in large ulcerated solid malignant tumours) in the future. This is very important for the potential treatment of tumours. The current status of knowledge and results of CAP application on gynaecological malign cell-lines or tissues are summarised in Table 1.

## 5. Plasma Physical and Chemical Characteristics and Plasma Sources in Medicine

Advancement in medicine was, for decades, characterised by the introduction of innovative technologies from physics to improve the diagnostic and therapeutic management of patients. From X-rays, magnetic resonance, nuclear medicine, PET-CT, and digital mammography to sophisticated radiation therapy (including intraoperative devices), all these technologies revolutionised medicine and brought enormous benefit for patients. In the last decade, a new form of technology is gaining relevance, bringing many opportunities for patient care, called physical plasma. Plasma is commonly known as the fourth state of matter (solid, liquid, gas, and plasma) [159]. Initially used for skin regenerative medicine [160], it is nowadays studied as regards anticancer treatment [27,28,161]. Depending on the plasma force, physical action is based on positive and negative ions, electrons, neutral atoms, photons, and electromagnetic fields, leading to the emission of visible ultraviolet (UV) radiation and thermal effects. 

Fundamentally, plasma consists of an ionised gas enriched with biologically and chemically reactive species, including charged electrons and ions, as well as radicals, atoms, and molecules in neutral (e.g., excited) or charged forms, where the electric charge can be positive or negative. In addition to chemical species, plasmas produce electromagnetic radiation, propagating disturbances such as shock waves and heating, among other effects. Medically relevant plasmas (termed CAP) benefit from low intensities of these individual effects, making them a gentle tool that can induce desired biological effects in a controlled manner [20]. CAP is generated under atmospheric pressure at ambient temperatures ranging from 20 °C to 50 °C [162].

Artificial plasma can be classified based on gas pressure (low-pressure vs atmospheric pressure plasma) or based on temperature (thermal/hot vs. nonthermal/cold plasma). Plasmas can be easily generated by applying an electric field to the process gas, typically pure helium or argon, or to a mixture including oxygen. This electric field accelerates electrons and initiates a cascade of chemical reactions that give rise to a diverse range of chemical species. The amount of applied energy and the type and pressure of the processing gas determine both the speed (and thus the temperature) and the chemistry of this cocktail of species. In medicine, low-temperature plasmas that can be generated at atmospheric pressure are desirable, due to the simplicity, versatility, and affordability of such plasma devices. 

Clinically, plasma-based electrosurgical devices have long been employed for blood and tissue coagulation, cutting, desiccation, and cauterising during surgery [163,164]. These devices involve heating tissue and their effects are primarily heat mediated. Recently, new sources of CAP with well-controlled temperatures below 40 °C have been designed and clinically applied in plasma medicine. The nature of direct plasma treatment renders it highly suitable for the treatment of primary tumours that arise from skin or mucosal surfaces. This technology may complement surgery as adjuvant therapy or specific therapy in combination with chemotherapeutics or radiation. Of particular clinical interest is the ability of CAP to penetrate tissues and effectively target cancer cells that have infiltrated healthy tissue adjacent to the tumour mass, and to eliminate micrometastases [161]. 

### Sources of Cold Atmospheric Plasma

New CAP sources used in plasma medicine can be classified into three types [162,165,166]:

1. Direct plasma sources: These plasmas use the human body (such as the skin, internal tissues, etc.) as an electrode. Thus, the current produced by plasmas has to pass through the body. The most commonly utilised technology in this category is the dielectric barrier discharge (DBD) plasma source. The major disadvantage of this technique is the application distance (between the electrodes) which must remain within a close range, generally less than three mm^2^, thus limiting its use for small areas of the human body [15].
2. Indirect plasma sources: These plasmas are generated between two electrodes. Active species that are created by the plasmas are subsequently transported to target application areas. Several devices are available, ranging from very narrow plasma needles or jets to larger plasma torches such as the kINPen^®^ MED, Atmospheric Pressure MicroPlasma Jet (APMPJ), InvivoPen, and MicroPlaSter^®^ α and β. Plasma jets can be classified according to parameters such as discharge geometry, electrode arrangement, excitation frequency or pattern.
3. Hybrid plasma sources: These plasmas combine the benefits of the two aforementioned plasma source types (e.g., using the plasma production technique of direct plasma sources and the essentially current-free property of indirect plasma sources). This is achieved by introducing a grounded wire mesh electrode, which has significantly smaller electrical resistance than that of the tissue. Thus, in principle, all current can pass through the wire mesh. The MiniFlatPlaSter is an example of a hybrid plasma source.

As a novel technology CAP expanded very quickly to several industrial and medical fields and rapidly increased its applications as a medical device or drug-mediated tool [167]. In biological applications, the most commonly used plasmas are atmospheric pressure plasma jets (APPJs) and dielectric barrier discharges (DBDs) [168].

Various types of APNP-Js with different configurations have been reported, where most of the jets are working with noble gas mixed with a small percentage of reactive gases, such as O_2_. Plasma jets operating with noble gases can be classified into four categories, i.e., dielectric-free electrode (DFE) jets, dielectric barrier discharge (DBD) jets, DBD-like jets and single electrode (SE) jets [169].

Several different gases can be used to produce cold atmospheric plasma, such as helium, argon, nitrogen, heliox, and air. Cold atmospheric plasma is created by many methods [170]. Each unique method can be used in different biomedical areas. A variety of different CAP devices have been developed and tested for research and clinical purposes. To date, four plasma devices have been certified for medical purposes. In 2013, the medical device kINPen^®^ MED plasma-pen (INP Greifswald/neoplas tools GmbH, Greifswald, Germany), an APPJ, and PlasmaDerm^®^ VU-2010 (CINOGY Technologies GmbH, Duderstadt, Germany), a DBD source, have been CE-certified in Germany by MEDCERT under the norm ISO 13485, and the InvivoPen system is used for laboratory conditions. The medical device SteriPlas plasma torch (Adtec Ltd., London, United Kingdom) was then certified for use in the treatment of chronic and acute wounds, as well as for reduction of microbial load [87,88,171,172]. Their great advantage, apart from favourable medical use, is their relatively low manufacturing costs [18], allowing for a reduction in the financial burden imposed on health budgets by conventional treatments.

The technology that brought CAP into medicine via experience with clinical applications for local disease control is currently intensively studied as a novel therapeutic agent in oncotherapy. Two methods of applying plasma are described: direct treatment and indirectly using PAM-nanoparticles and PAL (plasma-activated liquids). The first method consists of applying CAP directly to in vitro cells, in vivo animal models, or living human tissue. The second strategy consists of producing PAM and then applying (injecting) it into cell cultures or tumours. These approaches have been studied in recent years, and not only the number of cell lines-type studies, but in vivo studies based on animal models, human tissue medium, or clinically conducted on particular patients, proved its large anticancer potential, with advantages for patients suffering from malignancies [15,162,165,166]. 

## 6. Plasma Interaction with Human Tissue 

When CAP is applied, it induces both physical effects (production of ultraviolet rays, heat, and electromagnetic fields), and chemical effects (production of ROS/RNS = RONS). Whereas physical effects seem to have a negligible cellular impact, RONS may induce cell membrane alterations, lipid peroxidation, transient poor formation, alterations in protein structure, an increase in intracellular ROS/RNS, DNA double-strand brakes, and subsequently apoptosis (mitochondrial or cellular) [173], without causing thermal damage to the surrounding tissue [174]. Importantly, the source of plasma plays an essential role in cell/plasma interactions. Generally, it is accepted that low-dose plasma is associated with stimulation of processes such as cellular viability, the promotion of cell proliferation and migration. On the other hand, high-dose plasma leads to cellular apoptosis and necrosis, demonstrating apoptosis-independent anti-proliferative cell effects. Furthermore, a dose-dependent increase of cells observed in the G1 phase of the cell cycle indicates the important role of cell cycle regulation for anti-proliferative CAP mechanisms [175].

The first human-based tissue interactions with CAP were observed on fibroblasts and keratinocytes, which are two dominant cell types associated with wound healing, and that can be stimulated via CAP [176]. Ngo et al. (2014) [177] showed that atmospheric N2/Ar micro-plasma stimulated fibroblast proliferation and migration via the release of fibroblast growth factor-7. In another study, the authors used different plasma sources to stimulate keratinocytes. CAP activated molecules are also associated with angiogenesis in skin human epidermal keratinocytes, endothelial cells, and dermal fibroblasts [178]. Wound re-epithelisation after CAP intervention was also detected in a model of full-thickness acute skin wounds in rats [179]. In the same way, the use of N2/Ar plasma therapy to partial thickness skin wounds on murine [180] or mice [181] models resulted in wound healing promotion by altered keratinocyte and fibroblast migration, and changes in adherence junctions and cytoskeletal dynamics as shown by the downregulation of E-cadherin and several integrins, as well as actin reorganisation. The application of CAP on a diabetes model also revealed acceleration in wound healing accompanied by faster re-epithelialisation with the formation of a new epidermis layer, collagen deposition, less inflammation, as well as neovascularisation [182]. In vivo experimental models are now the next appropriate subjects to further analyse the positive impact of CAP on wound healing. There is a great need to address this issue as CAP could become an additional tool in vulva cancer surgery and postoperative management, especially among obese, immobile, or diabetic patients.

## 7. Plasma Promoted Wound Healing and Its Possibilities in the Surgical Treatment of VSCC 

Cutaneous wound healing is a complicated process involving various cells and cytokines. It is divided into an inflammatory, a proliferative, and a remodelling phase. Due to its complexity, it is easily affected by internal and external disturbances, which may lead to chronic or even non-healing wounds, causing serious medical problems [183]. Patients with chronic wounds have a poor health-related quality of life in general, and wound-related costs are substantial. As the prevalence of chronic wounds is greatly increasing [184], the development and implementation of wound management strategies that focus on increasing health-related quality of life and effectively reduce costs for this patient group are urgently needed. Here, CAP exerts its beneficial effects through various mechanisms. CAP may facilitate the transformation of a chronic wound from a stagnating wound to an acute healing wound, by inflammatory and proliferation supporting stimuli [185,186], including neovascularisation [187]. Some studies reported the positive effect of CAP on angiogenesis. ROS and RNS also belong among pro-angiogenic growth factors (e.g., VEGF, EGF, FGF, TGF) and cytokines (e.g., IL-1, 2, 6, 8; TNF). It seems that ROS/RNS may have an important role in wound vascularisation [188]. This is of enormous importance in patients with postoperative surgical skin flaps or site infection in vulva cancer patients suffering from comorbidities (e.g., obesity, diabetes, and vasculopathies). 

Although the trend of surgical treatment in vulvar cancer patients is towards less extended resections, a significant number of cases are still diagnosed with locally advanced diseases, requiring extended resections. The development of early and late postoperative complications following vulvar surgery is thus still a clinically important issue. Bacalbasa et al. (2020) [189] found that the risk of postoperative complications was significantly affected by: (i) the stage of the disease, (ii) the preoperative levels of serum albumin, (iii) the status of the resection margins, (iv) previous history of irradiation, (v) length of hospital stay, and (vi) the association of comorbidities. The most frequent complication was wound dehiscence, necessitating reoperation (21%), followed by urinary tract infection and lower limb lymphedema (both 17.3%). Authors indicate for the precise selection of cases submitted to surgery, which further supports the need for new therapeutic approaches and tools in the management of patients with vulva cancer. Once such complications occur, the first step of treatment is debridement to remove necrotic tissue and exudate, which is conductive to bacterial growth. Simultaneously, systemic or topical antimicrobial agents should be used to eliminate the extensive bacterial burden. The use of antimicrobial agents is often limited by hypersensitivity to antibiotics, however, and the increasing development of drug-resistant bacteria. Novel therapeutic alternatives to improve wound healing, especially on the vulva with problematic healing process are thus greatly needed. In view of all these complications, CAP has enormous potential to achieve a better postoperative outcome for patients.

As mentioned previously, CAP has a broad spectrum of medical applications due to its beneficial properties, including its antimicrobial effect, and the promotion of wound healing. Recent evidence has suggested that CAP intervention enhances the healing process via a reduction of the bioburden, and also via the stimulation of angiogenesis and production of skin cells. An antimicrobial effect was demonstrated in the early 1990s, leading to its application in the clinical sphere. CAP also has great potentials in regenerative medicine as a powerful tool for the treatment of chronic or acute wounds. The promising role of CAP as a medical approach has also been described in dermatology, including the impact of CAP on atopic dermatitis, pruritus, or psoriasis [190]. Several studies demonstrated the positive effect of CAP on the eradication of bacterial infection in chronic wounds associated with the promotion of healing processes [191]. Cold plasma successfully eliminated bacterial colonisation in patients with chronic leg ulcers [19], chronic wounds [192], or chronic venous leg ulcers [193], and resulted in enhanced healing of chronic wounds. Cold atmospheric argon was also observed to have a significant effect in patients with skin graft donor sites on the leg. Data revealed that cohorts of treated patients demonstrated better healing courses than placebo groups the second day after CAP intervention [194]. CAP has demonstrated a positive effect on skin grafts in leg surgery, and it would be interesting to find out whether the same benefit would be observed in vulva surgery, where skin grafts are commonly used after radical tumour resections. 

Metelmann et al. (2013) [195] analysed the effect of CAP in volunteers who had received ablative laser skin lesions. Experimental findings showed that the application of CAP promoted the inflammatory reaction necessary for tissue recovery in the early stage of the wound and also prevented posttraumatic skin disorders. There were no side effects of CAP associated with the development of precancerous skin lesions observed in tested individuals [195]. CAP was examined as a medical option for the acceleration of acute wound healing in a comparative study with different treatment groups (control, CAP, local treatment using betamethasone valerate ointment, and the application of basic fibroblast growth factor sprays). The results showed no significance between tested groups in wound healing; however, CAP demonstrated a more rapid recovery accompanied by a reduction in the redness and roughness of the skin. The authors observed no negative side effects from using cold plasma in the CAP group [196]. Recently, CAP was applied for the improvement of wound healing in different types of superficial skin erosion wounds, including patients with pyoderma gangrenosum, trauma wounds, giant genital warts, diabetic foot, and chronic eczema. According to data obtained from the different wound types, CAP accelerates wound healing through the eradication of bacterial colonisation, sterilisation of the wound, changing the local wound environment, and the promotion of tissue restoration [197].

These studies all demonstrated the significant clinical effect of CAP in healing processes with human subjects. The method was successfully used for pathogen eradication from both chronic and acute wounds via its biocidal effect. Evidence also suggests the beneficial role of CAP in the acceleration of healing different wounds without side-effects (i.e., premalignant lesions). As a result, CAP is an appropriate clinical approach for the treatment of wounds after surgical intervention, mostly for minimizing prolonged wound healing, which is associated with a poor prognosis due to delayed adjuvant therapy [198].

## 8. CAP Specific Abilities Predisposing Its Application in Anticancer Therapy

**Figure 1:**
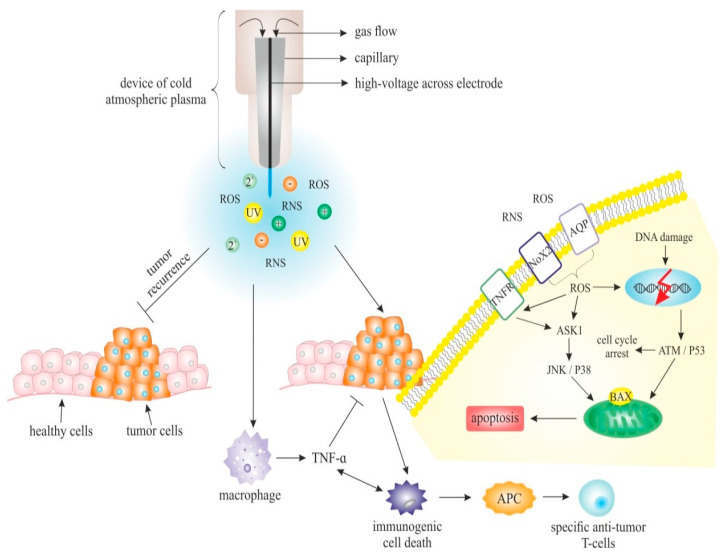
The mechanism of cold atmospheric plasma (CAP) in cancer treatment. Formation of plasma starts in high electric field across the region of gas (pure helium or argon, and/or their mixtures with oxygen) that accelerates electrons. These processes lead to the initiation of a cascade of chemical reactions associated with the generation of various chemical species. CAP is a source of highly reactive species (ROS, RNS, atomic oxygen, hydroxyl radical, superoxide, nitrogen oxides, and singlet delta oxygen), neutral particles (photons and neutrons), electrons, and physical factors (electromagnetic field and UV radiation) [199]. Reactive species produced by CAP have the ability to directly induce DNA damage and cell cycle arrest resulting in the apoptotic signalling of tumour cells. The production of reactive species can activate macrophages leading to higher elevation of TNF-α–mediated NF-κB activation and the expression of proinflammatory genes associated with tumourigenesis. On the other hand, CAP seems to be an effective inhibitor of TNF-α–mediated NF-κB activation with a potential role in anticancer strategies. CAP can also induce the immunogenic cell death (ICD) of tumour cells that lead to systematic immune response. ICD can also be achieved by the regulation of various cytokines, including TNF, that play a crucial role in the creation of immunogenic microenvironment [200]. Abbreviations: ROS, reactive oxygen species; RNS, reactive nitrogen species; AQP, aquaporin; TNFR, tumour necrosis factor receptor; Nox, NADPH oxidases; ATM, ataxia-telangiectasia mutated kinase; JNK, c-Jun N-terminal kinase; ASK, apoptosis signal-regulating kinase; APC, antigen-presenting cell; TNF-α, tumour necrosis factor alpha; Bax, Bcl-2-associated protein X; UV, ultraviolet radiation; APC, antigen-presenting cell.

It is generally accepted that CAP accelerates the healing of wounds with limited side-effects, and also has anticancer properties, and thus it would be very interesting to analyse the potential of its use in the treatment of both premalignant lesions and developed malignancies. The anticancer effects of CAP can be observed at several cellular or molecular levels (Figure 1), and can be briefly described as:

### 8.1. CAP Effect on Cellular and Extracellular Level

The expected basic cellular responses (apoptosis, growth inhibition, selective cancer cell death, cell cycle arrest, DNA and mitochondrial damage, a selective increase of ROS or immunogenic cell death) have been observed after the application of CAP to cell lines and/or tissue [28,201]. Several studies demonstrated the impact of CAP on different cellular processes associated with the suppression of cancer development via modulation of gene expression and other intracellular events [111,177,178,202]. Despite the current focus on CAP as a promising strategy for pathogen eradication contributing to wound healing, the exact mechanisms of the anticancer effect are not known [203]. Additionally, it is important to note that the interaction between plasma and tumour cells is essential, and the impact of plasma on the tumour microenvironment (TME) also plays a significant role in anticancer therapy [204].

Recent evidence revealed the effect of CAP on different compartments of TME (endothelial cells, immune cells, fibroblasts, collagen, fibronectin, elastin, proteoglycan, or glycoproteins). It has been reported that the prolonged application of CAP suppressed the collagen production and cell viability of murine fibroblast cells [205]. Similarly, a reduction of collagen secretion was demonstrated in keloid fibroblasts [206,207] after CAP intervention. 

The specific microenvironment of tumour cells causes different responses to increased levels of ROS and RNS, which subsequently leads to apoptosis [208]. Higher levels of cholesterol in plasmatic membrane are also typical for the majority of cancerous cells, and most notably for multidrug resistant cells. This is also accompanied by higher levels of phosphatidyl choline, phosphatidyl ethanolamine and phosphatidyl inositol, which makes the plasmatic membrane of these cells more rigid, and also less permeable for drugs. Conversely, the plasmatic membrane of metastatic cells possesses lower cholesterol, which makes them less rigid, and this facilitates these cells in entering the blood vessels [209,210]. Some studies suppose that lower levels of cholesterol in plasmatic membrane can be tissue specific in some types of breast and prostate cancer, regardless of their metastatic potential [209,210,211]. Importantly, lower levels of cholesterol in the plasmatic membrane of some tumorous cells also make the membrane more susceptible to peroxidation which results in higher pore formation, enabling the higher diffusion of ROS and RNS into the cell [28,202,212,213]. Lower levels of cholesterol are present only in a smaller portion of tumorous cells, but there are a few other features of the tumour microenvironment that are typical for wider variety of these cells, which means that CAP application results in the induces apoptosis. 

Another significant feature of the cancer microenvironment is the generation of superoxide anion O_2_^−^ into the ECM and the presence of protective catalase on the external surface of the cell membrane. The abundance of O_2_^−^ in the vicinity of the cancer cell membrane, which can be achieved by CAP application, triggers specific HOC1 and ONOO^−^ cell signalling pathways [214,215]. This subsequently leads to the formation of reactive OH radicals, lipid peroxidation and apoptosis. The presence of protective catalases associated with the external membrane can also be disrupted by CAP application [208,216]. A malignant cell microenvironment demonstrates higher activity in the proteasome complex involved in the degradation of intracellular proteins. It affects variable mechanisms in cancer cells, and it is also very significant in the regulation of apoptosis [217,218]. Proteasomes in malignant cells exhibited more sensitivity to the cytotoxic effect of their inhibitor compared to healthy cells, and the medical targeting of proteasomal activities thus became interesting for basic and clinical research [217,219,220].

### 8.2. CAP and Apoptosis

Apoptosis is the tightly regulated pre-programmed process of cell death essential for physiological homeostasis maintenance. The mechanism of apoptosis is regulated by caspases and occurs through two distinct molecular pathways. The extrinsic pathway is activated by the binding of extracellular death ligands, such as TNF, Fas-L, and TRAIL, to its death receptors. The intrinsic – mitochondrial derived pathway is initiated by intracellular stimuli and involves pro- and anti-apoptotic factors such as Bcl-2 proteins, cytochrome-*c*, and APAF-1 [221,222,223,224,225]. There has been strong interest in the targeted induction of apoptosis in recent years, as it is a very efficient non-invasive treatment [222,224,226]. 

Cold atmospheric plasma is also a potential targeted cancer treatment tool, as cancer cells are very sensitive to CAP-induced ROS [227]. Several studies have analysed different cell lines in apoptotic content. The loss of cell viability and shrinkage of tumours occurred mainly as a result of apoptotic processes, as evident from the specific morphological changes and higher activity of apoptotic cascade members [28,151,228,229].

A study of SiHa and HeLa cervical cancer cells treated by micro-DBD plasma revealed the different responses of one tissue type to CAP. SiHa cells had a significantly higher caspase-3 activity and thus lower survival rate, and a higher number of aberrantly expressed apoptosis-related genes compared to HeLa cells. CAP treatment also led to the alteration of 166 genes in the control fibroblast lines. The activity of caspases 6, 8, and 9 were similar in SiHa and HeLa cells. It was an interesting observation that CAP-treated cells entered them to the subG0 phase, both of cancer and fibroblast control cell lines [12]. 

Xia et al. (2019) [151] described the effect of ROS produced by CAP on the extrinsic apoptosis pathway members in A375 and A875 melanoma cell lines. Higher ROS dosage led to the overexpression of antagonistic protein SESTRIN 2, which resulted in the phosphorylation of p38 MAPK and increased expression of iNOS, FAS, and FASL. These changes triggered the activation of caspase 3 dependent apoptosis in the studied cell lines [151]. The increased activation and phosphorylation of JNK and p38 MAPK pathways was also observed after CAP application in HeLa cells [230], head and neck cancer cell lines [45], anaplastic thyroid cancer cell lines [231] and in vivo conditions in tumorous tissues in FaDu mouse xenograft models [45]. The CAP application resulted in the depolymerisation of mitochondrial membrane, accumulation of intracellular ROS and activation of caspase family protein. Similar results were also published by Kaushik et al. [5] in 2015 regarding altered phosphorylated ERK1/2/MAPK protein levels. They analysed various cell lines (MRC-fibroblasts, A549-lung carcinoma, T98G-glioblastoma, and HEK293-human embryonic kidney cells) and observed altered mitochondrial membrane potential and increased activation of caspase apoptotic mechanism. Apoptotic regulators located in the outer membrane of mitochondria, *BAX* and *BAK1* genes were upregulated. A higher expression of *H2AX*, a histone protein, with a phosphorylated form that can be considered a marker of DNA damage, was also observed. On the other side, *BCL-2* was downregulated in solid tumour cells. An increase of *BAX* and decrease of *BCL2* gene expression was also observed in breast cancer cell lines (MCF-7) treated by plasma and a combination of plasma and iron nanoparticles (NPs). The viability of cancer cells was significantly decreased and *BAX/BCL-2* ratio was altered in favour of apoptosis [227]. Yan et al. (2017) [27] also described the activation of apoptosis by ROS-stress response signalisation and regulation by BCL-2 protein family. CAP induced a sub-G(1) arrest in p53 wild-type OSCCs and increased the expression levels of ATM, p53, p21, and cyclin D1, confirming the involvement of DNA damage and triggering sub-G(1) arrest via the ATM/p53 pathway in the apoptosis mechanism [232]. Loss of viability, higher numbers of cell cycle arrests, and the increased activity of caspase 3 connected with a higher apoptosis rate after CAP treatment were observed in several cell lines, including HeLa, squamous carcinoma YD-9 cell lines and melanoma G361 cell lines, however, these changes were more significant in p53 mutated cell lines compared to wild type p53 cells [233].

Whether the application of CAP will initiate apoptotic or other processes depends to a large extent on the duration of exposure, distance, dose and duration of exposure and gas content. Low dose CAP treatment and an exposure less than 60 s leads to increased proliferation and wound healing, but a bigger dose and longer exposure time lead to controlled cell death [12,234,235,236]. Finally, known data indicates that CAP also seems to have a strong apoptotic effect on cancer cells resistant to current treatments. The mechanisms involved seem to depend to variable extents on p53, p38, NF-KB, JNK or caspase pathways [28].

### 8.3. CAP and Induced Gene Expressions, Proteomic and Epigenetic Changes 

CAP, with its anticancer effects, can induce DNA damage and cell cycle exit into senescence [166,229]. Welz et al. (2015) [237] demonstrated that CAP could decrease cell viability and increase DNA fragmentation leading to cell apoptosis. Furthermore, specific CAP-binding proteins and intracellular ROS can induce the expression of genes involved in cellular apoptosis mediated by TNFα and apoptosis signal-regulating kinase (ASK) [166]. The active genetic expressions with corresponding mRNAs transcriptions were also observed for genes encoding IL-12 (downregulation) and IL-1β, IL-6, IL-8, IL-10, TNFα, VGFR, and interferon-gamma (upregulation) after CAP exposition [217]. In vitro and in vivo studies aimed at wound healing also showed that plasma might induce the expression of IL-6, IL-8, MCP-1, TGF-b1, and TGF-b2 genes, which is crucial for the healing process [186]. The genomic impact of CAP is also demonstrated in the high selectivity for cell death and the removal of tumour cells from the proliferative phase of the cell cycle. Yan et al. (2015) [238] demonstrated that CAP increased the percentage of apoptotic tumour cells by blocking the cell cycle at the G2/M checkpoint, and this effect was mediated by reduced intercellular cyclin B1 and cyclin-depend kinase1, increased p53 and cyclin depending on kinase inhibitor and an increased Bcl-2-like protein4 (BAX)/B cell lymphoma2 (Bcl-2) ratio. Increased amounts of keratinocytes associated with the antiproliferative effects of CAP were also found in the G2/M1 phase [238]. 

The presence of reactive plasma species can also affect proteins and protein-based structures [239]. Protein modification is mainly initiated by ROS and RNS that can lead to etching, the cross-linking of proteins, oxidative reactions in protein building blocks, and cause the cleavage of proteins into peptides. Some studies report that functional groups such as carboxylic acid or amide bonds can be introduced to the surface of polymers. Tolouie et al. (2018) [239] demonstrated that CAP exposure can selectively alter the protein conformation and function, depending on biological origin, plasma type, and treatment conditions. Interestingly, the effect of CAP on enzymes is inconsistent. In some cases, CAP deactivates enzymes, whereas on the other hand, there are situations where CAP exposure led to increased enzymatic intracellular activity. The inactivation/activation of enzymes after plasma exposition depends on the ability of the cellular defence system to confront stress-induced situations [240]. 

It is known that CAP-activated media can mediate the anticancer effect on tumour cells. Utsumi et al. (2013) [75] described the effect of CAP-activated media for epithelial ovarian carcinoma cells. The aim of CAP exposure was the inhibition of tumour growth and promotion of apoptosis. CAP exposure can temporarily disrupt the cell membrane and affect intracellular signalling pathways. An interesting study by Schaner et.al (2003) [241] characterised gene expression in epithelial cancers of the ovary. This study showed that the most expressed genes in ovarian carcinomas were PAX8 (paired box gene 8), EFNB1 (ephrin-B1) and mesothelin. The study also revealed that numerous genes have different expression. The authors detected the overexpression of the transcription factor ATF3. The main role of ATF3 is to repress matrix metalloproteinase 2. The expression of ATF3 was higher in the ascites samples. The study also followed the expression of oestrogen receptor 1 and cytochrome P450 4B1. Their production was at relatively low levels in clear cell cancers, compared with other ovarian cancers. It is also interesting that E-cadherin was highly expressed and a member of the discoidin domain receptor family (DDR1) had a lower level of expression in clear cell cancers. It is known that NEAT1 (nuclear paraspeckle Assembly Transcript 1) is overexpressed in many cancers. Knutsen et al. (2020) [242] found that the level of expression of isoform NEAT 1-2 in human is higher upon lactation. This study also reported that the expression of NEAT1-2 correlated with HER2 (human epidermal growth factor receptor 2)-positive breast cancer. The role of NEAT1 is to regulate gene expression at both transcriptional and post-transcriptional levels. Recent studies reported that the loss of Zac1 expression is also associated with the progression of tumours, including cervical cancer, breast cancer and ovarian cancer. Su et al. (2020) [243] found that high Zac1 expression is associated with a poor prognosis of cervical cancer and with epithelial-mesenchymal transition. 

Several studies of breast cancer cell lines have reported promising results. Much data has provided evidence that epigenetic changes contribute to breast cancer progression Here, the DNA methylation pattern (induced hypermethylation at the promoter CpG sites) followed CAP application in a breast cancer cell line expressing the oestrogen receptor (MCF-7) [244]. MicroRNA miR-19a-3p (miR-19a) was identified as a mediator of the cell proliferation-inhibitory effect of CAP in the MCF-7 breast cancer cell. *ABCA1* and *PTEN,* which had been suppressed by miR-19a, recovered their expression through CAP treatment. CAP induced damage to DNA in the nucleus by producing a double-strand break (DSB). After exposure to CAP, these cells showed growth retardation, increased DSB, and apoptosis [38]. Many studies identified altered expression in cervical cancer. Another study reported an association between miR-218 expression and various clinicopathological features in cervical cancer. MicroRNA (miR) microarray analysis revealed that miR-218 is downregulated in cervical cancer tissues [245]. According to Su et al. (2020) [243], these results indicate that plasma induces epigenetic and cellular changes in a cell type-specific manner, suggesting that the careful screening of target cells and tissues is necessary for the potential application of plasma as a cancer treatment option.

### 8.4. CAP Induced DNA Breaks and Modifications 

It is known that the biological significance of DNA damage by RONS depends on the extent of damage, where it occurs in the genome, and how fast it can be repaired. As the damage of DNA has importance effects on replication and cell division, the CAP-induced RONS oxidative damage in strand breaks and chemical modification of DNA in the cancer cells leading to sub-lethal or lethal cell reaction is of interest [246]. Here, the advance of CAP is in its specificity to induce DNA strand breaks, surprisingly without any significant rupture of the phospholipid membranes [247]. The interest of studying CAP induced DNA changes is even greater, as cancer cells are more susceptible to the effects of CAP due to a higher percentage of cells in the S-phase of the cell cycle [248], and because CAP has demonstrated the ability to selectively ablate cancer cells while leaving healthy cells mostly unaffected [249]. 

The significance of damage to DNA by RONS depends on the extent of that damage, where the considerable DNA modifications and breaks usually halt cell replication and cell division. Arjunan et al. (2015) [250] observed that DNA mismatches in nucleobases induced by plasma irradiation can be genotoxic (can hydrolyse the N-glycosidic bond) and lead to cell death. Lackmann et al. (2012) [251] reported the expression of different gene fusions after treating cells with plasma in liquid culture and indicated that CAP emitted particles cause DNA strand breaks, whereas CAP emitted photons provoked cross-link DNA strands. Furthermore, DNA–protein crosslinks [252], DNA chemical modification 8-oxoguanine (8-oxoG), and the up-regulation of the 8-oxoG repair enzyme simultaneously with DNA strand breaks were induced after exposition to CAP [247]. 

The plasma-treated cells also show an accumulation of gamma-H2A.X, a known marker for DNA double-strand breaks, and higher p53 tumour suppressor gene activity as a response to DNA damage. Interestingly, cytochrome-related changes in mitochondria and its membrane augmented the CAP induced changes on a DNA level [253], and ROS and RNS lead to mitochondria-mediated apoptosis and to further activation of the DNA damage. The plasma effluents, and particularly the plasma-generated particles, also rapidly deactivated proteins in the cellular milieu. In addition to the physical damage to the cellular envelope, therefore modifications to DNA and proteins contribute to the anticancer and anti-bactericidal properties of cold atmospheric-pressure plasma [254]. 

### 8.5. CAP and Induced Redox ROS and RNS Effect

As studies have demonstrated that CAP can induce apoptotic cell death in cancer cells, determining the plasma effect on them is a crucial issue. CAP effects on in vivo or in vitro structures, as indicated previously, are mediated by biologically active factors such as the electric field, charged particles (ions and electrons), photons and UV radiations, free radicals, and reactive oxygen and nitrogen species (RONS) [229]. CAP exposure induces redox effects ROS (reactive oxygen species) and RNS (reactive nitrogen species) in cells or tissue, where these reactive species act as antimicrobial molecules produced from nitric oxide and superoxide, causing nitrosative cellular stress. Both ROS and RNS are “double-edged swords”, and most atmospheric pressure plasma jet (APPJ) applications focus on the oxidative and/or nitrative stress on bacteria, cells, and tissues [255]. ROS/RNS modulates numerous redox-sensitive biochemical pathways in physiological and pathophysiological cellular processes, affecting cellular integrity. Such induced oxidative modification of biologically essential molecules leads to their functional impairment, such as the loss of biological membranes and structural proteins [256]. At the cellular level, ROS can regulate protein phosphorylation, ion channels activity, and transcription factors involved in critical biosynthetic processes [257]. As the antioxidant mechanism in cancer cells is low, contrary to healthy cells, the RONS-mediated selective effect of CAP mostly affects cancer cell viability. Here the molecular level responses to ROS are related to both redox and phosphorylation signalling with proteins [6].

The biological mechanism of the CAP-induced RONS effects on cells can be explained in two ways. The first involves the insertion of hydrogen peroxide (H_2_O_2_) to a ROS regulation system. The second involves the changes in mitochondrial transmembrane permeability induced by RNS [258]. The effect of RONS is thus harmful for cells in both its functional and structural being. ROS can damage mitochondrial DNA and cause changes in the permeability of transition pores in mitochondria, which leads to the induction of apoptosis. The most harmful ROS are superoxide anion (O_2_^−^), hydrogen peroxide (H_2_O_2_), and hydroxyl radical (OH) [259]. Superoxide and nitric oxide have a role as physiological signalling messengers. Hydrogen peroxide has been suggested as the most crucial signalling messenger in vivo [260]. The generation of ROS begins with the rapid uptake of oxygen, activation of NADPH oxidase, and the production of the superoxide anion radical. The O_2_^−^ is then rapidly converted to H_2_O_2._ H_2_O_2,_ is further converted to hypochlorous (HOCl) a potent oxidant and antimicrobial agent. Superoxide is removed by superoxide dismutase (SOD), and singlet oxygen is quenched by carotenoids [256]. Under physiological conditions, O_2_^−^ and H_2_O_2_ appear incapable of directly causing strand breaks or nucleobase modifications in DNA [250].

The regulation-impaired effect of ROS can be explained by the impact on various processes such as proliferation, metabolism, differentiation, and survival, and also by regulating redox-reactive residues on proteins. Most regulators of redox signalling are members of the thioredoxin (Trx)-fold family of proteins. TRX fold proteins, such as thioredoxins (Trxs), glutaredoxins (Grxs), and peroxiredoxins (Prxs), have been characterised as electron donors, guards of the intracellular redox state, and “antioxidants”. Today, these redox catalysts are increasingly recognised for their specific role in redox signalling [261].

In today’s medicine, RONS has a role in many therapies, including oncology, dermatology, and dentistry. Plasma treatment gives us an opportunity to modulate the healing process and therapeutic response in target cells and tissues.

## 9. CAP as a Novel Anticancer Treatment Modality, Including Vulvar Pathologies

The use of plasma in the treatment of vulvar pathologies is not unknown. It is not CAP, however, but plasma argon beam coagulation that is used to treat, for example, multifocal VIN III lesions with a favourable clinical outcome. It helped to successfully treat (51.7%) patients with this diagnosis, and no recurrence was demonstrated within the follow-up period of 34.9 months [262]. This experience with plasma medicine in oncogynaecology and positive results from CAP-associated studies in general, is therefore promising for plasma treatment in vulva cancer, which can be used as follows: (a) local induction of immunogenic cell death; (b) induction of cellular immune memory; (c) induction of system response against cancerous cells [263]; (d) surgical removal/reduction of the tumour; (e) elimination of micrometastases through cancer-selective cell killing; and (f) improved chronic wound healing (mainly via antibacterial effects), supporting palliative care.

Consequently, there is increasing interest in oncology-focused research in the application of CAP in anticancer treatment. As shown in Table 2, scientists are now intensively focused on the direct or indirect (via the use of PAM or PAL) CAP effect on both gynaecological or non-gynaecological cancer types, and on the synergic use of CAP and nanotechnology, as well.

### 9.1. Direct Anti-Tumour Effects of CAP

The direct anti-cancer efficacy of CAP is mainly associated with the treatment of tumours that arise from skin or mucosal surfaces [274]. Importantly, direct CAP treatment is related to the higher cytotoxicity of activated cells due to the reactive species produced by CAP. In comparison with the indirect effects of CAP, the activation of cells is suggested to be mediated by short-lived reactive species or other unknown factors [275]. The direct anticancer effects of CAP are associated with ROS and RNS, where ROS is closely related to calcium signalling. The second messenger is calcium involved in various cellular processes, for example, tumourigenesis, apoptosis or senescence. Its cellular influx can by modified by CAP, as was demonstrated by Schneider et al. (2018) [264] using a specific device developed for the treatment of cancer cells and tissue in solution (phosphate buffered extracellular solution) exposed to CAP, triggering the senescence of melanoma cells [264] and increasing cellular pH acidity [30]. Xia et al. (2019) [151] described previously unrecognised mechanisms of melanoma cells responding to CAP treatment in PBS via an induction of apoptosis of melanoma cells through Sestrin2-mediated nitric oxide synthase signalling. CAP generated with argon gas and exposed to cells in a cultured medium can, in a dose-dependent manner, modify a pair of genes and their antisense lncRNA expression, leading to an antiproliferative effect in breast cancer cells [265] or the decreased mobility of ovarian cancer cells exposed directly to the CAP or to cell culture medium [75], and severe strand breaks with chemical modifications of their intracellular DNA induced by the plasma irradiation of lung cancer cells treated in medium [247]. The application of CAP thus opens novel opportunities for cancer treatment [75]. The medical application of CAP requires more in-depth knowledge about its molecular background [264] of gaining biological activity and also the potential emission of harmful noxaes [274]. 

### 9.2. Indirect Anti-Tumour Effects of CAP

More recently, the unique chemistry of CAP was demonstrated to transfer and to be retained in plasma-treated solutions, also known as “plasma-activated media (PAM)”. This significantly expands the scope of the potential application of CAP technologies to those cases where tumours are, for example, hard to reach, or where there is a need for selective cell killing. Here, PAM is a valuable help in enhancing the efficacy of traditional chemotherapy agents [273]. CAP acts as a supportive tool, inducing chemical species and electric fields for better drug delivery to targeted cellular or molecular structures.

PAM may be a promising tool in cancer treatment with the formation of O_3_ as a probable mechanism, as was demonstrated by the analysis of cancer cell lines, of which human breast cancer cell line SKBR3 was most susceptible to PAM [266]. In vitro, and in vivo analysis also revealed that PAM could selectively trigger apoptosis, and hinder the proliferation and migration of triple-negative breast cancer compared to the other subtypes [55]. PAM inhibited the cell migration, invasion, and adhesion of ES2 cell in vitro and suppressed metastatic potential in an in vivo model of intraperitoneal metastasis [157]. 

Plasma-activated liquids may be a novel therapeutic approach to the treatment of peritoneal metastasis in gastric cancer, as was demonstrated by the attenuation of gastric cancer cells migration and adhesion in vitro by PAM and a decrease in the formation of peritoneal metastatic modules in a mouse model in vivo [267]. The future application of the anti-cancer capacity of the cold plasma-stimulated medium can also be utilised through its stabilisation during storage at 8 °C and −25 °C for at least three days using PBS and cysteine/methionine-free Dulbecco’s Modified Eagle Medium [276]. Similarly, plasma-treated phosphate-buffered saline (pPBS) was demonstrated to be more stable in practical clinical application due to its higher solubility in comparison with PAM [277]. 

An injection of PAM can be used to treat the superficial tumours, including vulvar cancer, by a direct approach, which opens a new path in cancer treatment and a path for new pharmaceutical products. Furthermore, treatment with CAP can also be undertaken directly or indirectly due to the anatomical position of the vulva cancer. 

### 9.3. Dual Cancer Therapeutic Approach: Synergy of CAP and Nanotechnology

Cold atmospheric plasma is an emerging biomedical technique that shows great potential for cancer treatment in a novel dual cancer therapeutic method by integrating promising CAP and iron oxide-based magnetic nanoparticles (MNPs) for targeted cancer treatment. Li et al. (2019) [91] showed that the effectiveness of CAP and iron oxide-based MNPs for synergistic application aggressively killed activity against lung cancer cells, and significantly inhibited cell proliferation via a reduction of viability and induction of apoptosis. Importantly, combining CAP with iron oxide-based MNPs induced EGFR downregulation, while CAP inhibited lung cancer cells via depressing pERK and pAKT. The translation of these findings to an in vivo setting demonstrates that CAP combining iron oxide-based MNPs is effective at preventing xenograft tumours. The integration of CAP and iron oxide-based MNPs is thus a promising tool for the development of a new cancer treatment strategy with a significant shift in the current paradigm of cancer therapy [91]. A dual cancer therapeutic method based on the integration of CAP and novel drug-loaded core-shell nanoparticles for the targeted treatment of breast cancer also revealed the synergistic inhibition of cancer cell growth, down-regulation of metastasis-related genes and facilitation of drug-loaded nanoparticle uptake with potential benefits in minimising drug resistance [40]. 

CAP and silymarin nanoemulsion activated autophagy in human melanoma cells by activating the PI3K/mTOR and EGFR pathways and modulation of the expression of transcriptional factors and specific autophagy-related genes [268]. Co-treatment with PEG-coated gold nanoparticles and CAP also inhibited the proliferation of cancer glioblastoma and lung adenocarcinoma cells through blockading the PI3K/AKT signalling axis, and reversed epithelial-mesenchymal transition (EMT) in solid tumours, thus preventing the growth of tumour cells, which was also observed in vivo [269]. A synergy of CAP and nanoparticles was found to be a promising approach in the therapy of colon cancer as was demonstrated by increased cell death in the presence of gold nanoparticles [270], and helium-based CAP-induced ROS-mediated apoptosis was attenuated by platinum nanoparticles in human lymphoma cells [271]. He et al. (2020) [48] demonstrated the ability of CAP to stimulate clathrin-dependent endocytosis to repair oxidised membrane and promoted the uptake of gold nanomaterial in glioblastoma multiforme cells. Similarly, direct exposure to CAP activates gold nanoparticle-dependent toxicity through an increase in endocytosis and trafficking to lysosomes in the same cell line [272]. A novel dual cancer treatment approach characterised by paclitaxel-loaded core-shell magnetic nanoparticles and CAP has potential as a tool for cancer treatment strategy, as was demonstrated by the growth inhibition of non-small cell lung cancer cells in vitro [273]. 

Above all, the integration of CAP and nanoparticles is a promising tool for the development of novel cancer treatment strategies. A synergy of nanoparticles, characterised by improved biocompatibility, lower cytotoxicity, and efficacy, and CAP that was explored to selectively target and kill cancer cells represents a new paradigm for a targeted cancer therapeutic approach [14]. Table 2 shows an overview of current trends in the anticancer potential of CAP mediated directly, indirectly, or via synergy with nanoparticles.

### 9.4. Immunotherapy and CAP

Current trends in the establishment of CAP as a robust approach in anticancer therapy are also associated with the synergic use of plasma and nanotechnology or its application in the modulation of an immune response. The human immune system is significantly associated with cancer, and the ability of the immune system̕ s modulation could overcome the capacity of cancer cells to suppress immune responses through several mechanisms, including cytokines, cell-based therapies, immune checkpoint blockades, and immunogenic cell death [228]. As immunotherapy has become an essential part of anticancer treatment, the CAP effect on the immune system was evaluated in several studies. For example, plasma-activated liquid media (PALM) rich in H_2_O_2_ reduced proliferation and increased calreticulin exposure and ATP release in pancreatic cancer cells, suggesting its potential to induce immunogenic cell death through activation of the immune system [278]. Van Loenhout et al. (2019) [279] showed that CAP-treated pPBS (plasma-treated phosphate-buffered saline) had the potential to induce immunogenic cell death, and eliminated the immunosuppressive tumour microenvironment in pancreatic cancer cells. pPBS treatment led to the more immunohistomodulatory secretion profiled defined by higher TNF-α and IFN-γ, lower TGF-β in coculture with dendritic cells [279]. These results offer a strong basis for further in vivo evaluation, which is actually partially studied in a clinical setting. Until now, CAP has been applied as an adjunct to immunotherapy in the treatment of glioblastoma multiforme due to its ability to upregulate the immune system by ROS induction [90]. 

Kaushik et al. (2019) [280] noted another positive effect of plasma on the immune system. They report that plasma treatment stimulated the differentiation of pro-inflammatory (M1) macrophages to a greater extent. This stimulated macrophages to favour anti-tumourigenic immune responses against metastasis acquisition and cancer stem cell maintenance in solid cancers in vitro. The differentiation of monocytes into anticancer macrophages (particularly increasing numbers of mitochondria and lysosomes) could also improve the efficacy of plasma treatment, especially in modifying the pro-tumour inflammatory microenvironment by affecting the highly resistant immunosuppressive tumour cells associated with tumour relapse. [280].

Recently studied plasma-related immunogenic effects include the use of CAP-mediated immune checkpoint blockade (ICB) therapy integrated with microneedles (MN), described for the transdermal delivery of ICB by Chen et al. (2020) [89]. They found that a hollow-structured MN (hMN) patch facilitated the transportation of CAP through the skin, causing tumour cell death. The release of tumour-associated antigens promoted the maturation of dendritic cells in the tumour-draining lymph nodes, subsequently initiating a T cell-mediated immune response. Anti-programmed death-ligand 1 antibody (aPDL1), an immune checkpoint inhibitor, released from the MN patch further augmented antitumour immunity. Their findings indicate that the proposed transdermal combined CAP and ICB therapy can inhibit the tumour growth of both primary tumours and distant tumours, prolonging the survival of tumour-bearing mice [89].

## 10. Advancements in VC Therapy Based on Better Profiling and Novel Technologies Combining CAP with Existing Treatments

The shift to a modern treatment of vulva cancer is becoming evident, as molecular targeted approaches, evaluated either in monotherapy or as potentiators of chemotherapy, are now widely studied in clinical settings. Wang et al. (2018) [281] found out that the deregulation of CHK1 function often occurs in VSCC and might contribute to tumourigenesis. Targeting CHK1 might thus be a useful antitumour strategy for the subgroup of VSCC harbouring p53 mutations, which is a common finding in VSCC, as well as other genes (NOTCH1, HRAS, and CDKN2A mutations) [282,283], which may activate the PI3K/AKT/mTOR pathway, thus, providing a rationale for new anti-VSCC therapies targeting this signalling actionable pathway. This search for a novel therapy is confirmed by increasing numbers of studies. Brunetti et al. (2017) [284] studied the juxtaposition of two different genes or gene parts due to chromosomal rearrangement, which is a well-known neoplasia-associated pathogenetic mechanism, and found two recurrent fusions with STIP1-CREB3L1 and ZDHHC5-GPR137 present in VSCC. The transcripts were detected only in the tumour samples, not in normal vulvar tissue from healthy controls. They supposed that the detection of such tumourigenic fusions might serve as therapeutic targets for antioncogenic drugs that interact directly with the molecular changes responsible for neoplastic transformation of VSCC. The importance of a molecular approach in VSCC carcinogenesis is also demonstrated by Agostini et al. (2016) [285], who revealed downregulation of the fragile histidine triad (FHIT) and upregulation of the high mobility group AT-hook 2 (HMGA2) gene via miR-30c and let-7a. The results of such translational research involving the molecular landscape of vulva cancer in the past was the basis for today’s clinical target-directed therapeutic agents: for example, erlotinib, an inhibitor of the tyrosine kinase activity of the epidermal growth factor receptor (EGFR); bevacizumab, a monoclonal antibody targeting the vascular endothelial growth factor (VGFR); and pembrolizumab, an inhibitor of the programmed death-1 interaction with its ligand, called the PD1-PD-L1 immune checkpoint used in immunotherapy of VSCC [286,287,288,289]. 

As vulva cancer is a very particular malignancy, where most cases gain exceptional inherent radioresistance after standard therapy (surgery, chemo/radiotherapy) [290], the development of other forms of additional treatment would be more advantageous. 

Here, the clinical approach is to use electrochemotherapy as a feasible, easy to perform, and reproducible procedure in patients with primary or recurrent vulvar cancer who are unable to undergo surgery. Survival after one year in this population was observed at 50%. Electrochemotherapy may have a role in the management of vulvar cancer, especially as a palliative treatment when other therapies are no longer applicable [291]. However, technical issues mean that this tool is sporadically applied. Conversely, CAP has received much more attention from researchers due to its ability to specifically induce the selective death of cancer cells over normal healthy cells, called antitumour selectivity [291,292,293], by activating the apoptosis of these cells, decreasing their proliferation and mobility, and by recovering their sensitivity to therapeutic drugs. Previous studies showed encouraging results where CAP returned cisplatin-resistant ovarian cancer cells [75], paclitaxel-resistant breast cancer cells [158], tamoxifen-resistant breast cancer cells [39], and temozolomide-resistant malignant glioma cells [294] to a drug-sensitive state, in addition to inducing apoptosis and growth inhibition. CAP showed enhanced sensitivity to radiation by generating ROS with a simultaneous inhibition of tumour growth [295]. Selectivity in the efficient killing of cancer cells (plasmas self-adaptive toward cancer cells) [296], without adverse toxicity to healthy cells/tissues (as commonly present in radiation therapy), will be one of the most important therapeutic considerations in assessing CAP as a new cancer therapeutic strategy alone or combined with ionising radiation or chemotherapy. These findings may contribute to extending the application of CAP to the treatment protocols in cytotoxic drugs or radiation therapy-resistant cancers. Such effect was previously enhanced by a static magnetic field [297].

CAP was also studied to enhance photodynamic therapy (PDT), which is a non-invasive method for the treatment of superficial malignant cancers. The known limiting challenge of PDT is the hypoxic conditions during treatment, which reduce PDT efficiency. ROS and free radicals in the plasma flame output demonstrated that CAP could improve treatment efficiency of both indocyanine green (ICG) and protoporphyrin IX (PPIX) in breast and colon cancer cell lines [298]. This offers a promising background for conducting future studies in vulva cancer application, for both superficial lesion treatment and ICG guided nodal sampling.

Cold atmospheric plasma has been proposed as a novel therapeutic method due to its anti-cancer potential in combination with hyperthermia (HT) 42 °C or radiation 5 Gy. Synergistic enhancement in cell death with HT and an additive enhancement with radiation were observed following helium-CAP treatment. These findings would be helpful when establishing a therapeutic strategy for CAP in combination with radiation or HT [299], and specifically also used in ovarian cancer patients intraoperatively [300]. CAP application in an in vivo mouse model of intraperitoneal ovarian cancer metastasis via PAM model inhibited the peritoneal dissemination of cancer cells, resulting in prolonged survival [157]. 

The recurrence of cancer due to the acquisition of chemoresistance to ‘classical’ cytotoxic chemotherapeutics and molecularly targeted therapies [301] or radioresistance [302] remains a severe problem for the clinical treatment of cancer patients. A relatively high number of cancer patients receiving chemotherapy develop recurrent or metastatic disease due to acquiring drug resistance over time through different mechanisms, including multi-drug resistance, cell death inhibiting (apoptosis suppression), alterations in the drug metabolism, epigenetic and drug targets, enhancing DNA repair and gene amplification [303,304]. 

The approaches and research activities noted above confirm the importance of significant molecular and technical advancement for further clinical use in the management of patients with vulvar cancer or its premalignant lesions. This “paradigm shift” from surgery with chemo/radiotherapy alone to novel diagnostic, preventive and therapeutic approaches is well supported by the strong preventive and therapeutic potential of HPV vaccines, and is a promising therapeutic response by treatment of VIN [305].

## 11. A paradigm Shift from Reactive to Predictive, Preventive and Personalised Medicine (3PM)—Prominent Examples in the Context of Vulva Cancer and Premalignant Lesions

As already explained above, vulva cancer is an excellent example of implementing the paradigm shift from reactive medicine (defined as disease care) to cost-effective and patient-friendly 3PM approaches (healthcare) [306]. To this end, it has to be emphasised that the patient stratification based on individualised profiling plays a crucial role in 3PM strategies [307,308]. The levels of patient stratification are classified below, providing prominent examples of3PM implementation in the area.

### 11.1. The Primary Level of Targeted Prevention

HPV vaccination was mentioned above as one of the prominent approaches in preventing vulvar cancer linked to HPV lesions. In contrast to the human papillomavirus as a possible trigger of the disease, the role of vulvar-vaginal dryness as an essential risk factor is greatly underestimated in currently applied diagnostic and treatment approaches as demonstrated by Olga Golubnitschaja and colleagues [309,310]. At this level of targeted prevention, the main focus is on people in suboptimal health predisposed to the disease development (latent chronic VIN pathologies). The most prominent example is demonstrated for individuals with Flammer syndrome (FS) phenotype, with a higher prevalence in young female populations, and academicians demonstrating signs and symptoms of primary vascular dysregulation, a tendency to perfectionism, strong stress sensitivity, and healing impairments, amongst other things [310]. A topic-dedicated study demonstrated the FS phenotype as potentially characteristic for premenopausal females with vulvar-vaginal dryness [309]. Specifically, in this patient cohort, excessive vasoconstriction, feeling cold, low blood pressure, dizziness, strongly reduced thirst perception, strong smell perception, headache, perfectionistic personality, and tinnitus have been demonstrated as frequently co-exhibited symptoms [309]. Since many of the risk factors linked to FS carry a clearly preventable character, this phenotype is of great clinical utility for screening programs, in order to prevent female genital cancers, which may occur at any age. Contextually, individualised profiling is instrumental for mitigating measures tailored to the person suffering from vulvar-vaginal dryness as part of Sicca syndrome in individuals with the FS phenotype. 

### 11.2. The Secondary Level of Targeted Prevention 

The secondary level of prevention deals with complications linked to clinically manifested pathologies such as impaired wound healing, which is one of the prominent examples in this article that is treatable by CAP. If not diagnosed and treated well in time, delayed and/or impaired healing may cause chronic inflammation and cancer development, amongst other problems [311,312]. In addition to non-modifiable risks such as advanced ageing, there are many easily preventable factors involved in impaired healing, such as suboptimal lifestyle and nutritional and vascular deficits [313]. Taking into consideration a highly heterogeneous cohort of patients suffering from impaired healing, individualised profiling as a predictive diagnosis is instrumental for cost-effective targeted secondary prevention, as demonstrated in the multi-professional publication “Wound Healing: Proof-of-Principle Model for the Modern Hospital—Patient Stratification, Prediction, Prevention and Personalisation of Treatment” [314].

### 11.3. The Tertiary Level of Targeted Prevention

At this level, mitigating measures are applicable, for example, to avoid metastatic disease in vulva cancer patients. As described above, CAP application is of great importance in protecting the patient against local metastatic spread. To estimate the potential for metastatic spread to distanced organs, however, liquid biopsy is instrumental in predictive diagnosis at this stage of cancer progression [315]. To this end, CTC enumeration and the identification of highly specific multiomic patterns in the blood (e.g., miRNAs, CpG-changes, cfDNA) are considered an optimal approach [316,317] followed by personalised chemoprevention [318] and/or targeted therapy [319]. 

## 12. Status Quo and Clinically Relevant Perspectives

The application of cold physical plasma in a medical setting is rapidly increasing. It is a well-established therapeutic approach in dermatology, and CAP application in head and neck cancer patients is moving upwards in the pyramid of evidence-based medicine (EBM). The effectiveness of plasma in cancer cell lines, cultivated human tumour cells, human tumour specimens freshly explanted from patients, animal model tumours, and animals with transplanted human tumour stem cells is well documented. There is consensus among experts, referred to as EBM-level IV, regarding the response to plasma in experimental settings and proof of concept by clinical pilot studies, EBM-level III, for plasma treatment of head and neck cancer. It is already a concept in the palliative care of patients with locally advanced head and neck cancer and contaminated ulcerations because of proven effectiveness against microbial pathogens. Patients greatly appreciate that plasma reduces the strong fetid odour and pain, and is not accompanied by severe side effects [320]. 

The potential for the extensive clinical use of CAP is also significant in other malignancies, including oncogynaecology. An ongoing trial (NCT02658851, Florida, USA) is assessing the effect of CAP on the reduction of lymphocele following pelvic lymph node dissection during robot-assisted radical prostatectomy [321] (which is also a common procedure in cervical, uterine or ovarian cancer surgery), based on the authors’ previously noting the high incidence of this pathology [322]. Another ongoing clinical trial (NCT03218436, Tübingen, Germany) is studying the effect of CAP on human cervical neoplasia with histologically confirmed CIN 1-2 lesions [323], as a consequence of proof-of concept studies on cervical tissue from human donors [69]. Both studies have strong potential for oncology. The concept that the anticancer potential of CAP affects the dysplastic cells will shortly be of particular interest, including the treatment of vulvar intraepithelial lesions. Plasma is thus regarded as a potential intraoperative and adjuvant therapy. Its therapeutic efficacy should, therefore, be assessed in combination with current treatment strategies, mostly utilising PAM nanoparticles in maximising therapeutic effect and overcoming radioresistance when applied directly against tumour [295,324]. However, before a specific clinical application of CAP on vulva cancer, animal models are needed to stratify this conclusion in a more biologically relevant system, and several technical parameters need to be solved (e.g., penetration depth, optimal dosage, repetitive applications, type of CAP source device), and medical protocols created in line with safe clinical practice. 

Additional perspectives should involve:

(a) Precise drug-directed studies on chemoresistant, hormone resistant or radioresistant cancer cells in single or repetitive CAP applications.

(b) Studies aiming to use CAP as an adjunct tool during intraoperative resections or adjuvant chemotherapy, and as a potential tool against micrometastases outside surgically removed tumours.

(c) Studies using CAP during the palliative care of large inoperable metastatic cancer.

(d) Research aiming to achieve better control for surgical margins sufficiency.

(e) Studies evaluating CAP as an intraoperative tool for local groin lymph nodes silencing in patients undergoing sentinel lymph node biopsy alone, and omitting ipsilateral or contralateral inguinal nodal dissection.

(f) Studies in precision medicine using disease-optimised ROS cocktails via specifically engineered plasmas.

The first clinical case reports should then be conducted in locally advanced stages among patients with palliative care, and positive outcomes should motivate further clinical trials to demonstrate the relevance of CAP in clinical practice for patients with vulvar cancer (basal cell carcinoma, squamous cell carcinoma, malignant melanoma) and its premalignant lesions.

## 13. Conclusions 

Extensive research has been focused on the surgical and adjuvant management of vulvar cancer in the past and huge efforts on deciphering the molecular mechanisms of VSCC carcinogenesis. Technical tools are now providing increasing knowledge, having previously been sporadically applied in oncology. The potential for the extensive clinical use of CAP in oncogynaecology is immense, as CAP has been shown to be successful in various medical applications. Plasma is a potential intraoperative and adjuvant therapy, as intense preclinical studies have demonstrated the unique traits of plasma oncotherapy, such as its multimodal activity, synergic interactions with conventional chemotherapy agents, ability to cause genetic, epigenetic changes affecting processes fundamental to cancer progression and capacity to induce immunogenic cell death. Its therapeutic efficacy should, therefore, be assessed in combination with current treatment strategies (surgery, chemo- or radiotherapy), mostly utilising PAM nanoparticles in maximising therapeutic effect and overcoming radioresistance when applied directly against tumour. 

Before the real clinical application of CAP on vulva cancer, however, animal models are needed to stratify this conclusion in a biologic relevant system, and several technical parameters need to be solved (e.g., penetration depth, optimal dosage, repetitive applications, type of CAP source device and administration), and subsequent medical protocols created in line with safe clinical practice. Feasible groups of patients for such clinical trials are those with cancers lacking an effective targeted therapy, tumours that resist radiotherapy, cancers with physical isolation, patients with a relapse of metastases, postoperative patients, cancers that require low penetration depth (e.g., melanoma, skin, vulvar) and cancers with aesthetic requirements (often present with vulvar cancer and skin flaps). This review information may, in the future, serve as a foundation for the design of clinical trials to assess the efficacy and safety of CAP as adjuvant therapy for vulvar skin cancer.

Future strategies in the area could consider highly protective and cost-effective 3PM approaches comprising individualised profiling, predictive diagnosis, innovative screening programmes focused on young populations and individuals in suboptimal health conditions, targeted prevention, and treatments tailored to the individual [309,314,315,325]. 

## Figures and Tables

**Table 1:** Overview on available studies of cold atmospheric plasma (CAP) in gynaecologic cancer cell lines.

Cell Line Origin	Cell Line/s	Main Effects of CAP on Cell Lines Observed in the Studies	Ref.
Cervix	HeLa SiHa HFB	° Reduced viability of cells after plasma treatment in a dose-dependent manner ° Selective inhibition of proliferation in cancer cells compared to HFB ° Higher inhibition effect in the case of SiHa cells in comparison to Hela cells ° Significant increase of cells in subG0 phase cell and vice versa: reduction of populations in S phase and G2/M phase in a cell-type-specific manner ° Identification of caspase-3, -8 and -9 activation as an important mechanism underlying apoptosis in plasma-treated cells	[12]
Cervix	HeLa HFB detroit551	° Induction of HeLa cell apoptosis by facilitating an accumulation of intracellular reactive oxygen and nitrogen species (RONS) in a dose-dependent manner by both dielectric barrier discharge (DBD) plasma and nitric oxide-plasma activated water (NO-PAW) ° Higher selectivity of NO-PAW at given conditions	[62]
Cervix	HeLa	° Inhibited proliferation and induced cell death in an exposure time-dependent manner ° Significant suppression of the migration and invasion ° Reduced activity and expression of the matrix metalloproteinase (MMP)-9 enzyme ° Decreased phosphorylation level of both ERK1/2 and JNK, but not p38 MAPK	[63]
Cervix	CaSki DoTc2-4510 SiHa C-33-A	° Time- and energy-dependent effects of the treatment on cell proliferation ° Higher sensitivity of cervical cancer cells to plasma treatment in comparison to non-cancerous cervical tissue cells ° Decreased metabolic activity in cancer cells lines when compared to NCCT	[64]
Cervix	CaSki	° Distance and flow rate-dependent effect of CAP on tumour cell viability ° Dose-dependent induction of tumour cell death by CAP treatment	[65]
Cervix	HeLa	° Augmented number of early apoptotic cells, late apoptotic cells, but rarely necrotic cells by treatment with N2 and air plasma jets ° Induced apoptotic cell death in a dose-dependent manner ° Increased level of ROS and consequently, induction of apoptosis ° Induction of the mitochondria membrane depolarisation, causing increased mitochondrial transmembrane permeability and release of proapoptotic factors ° Blocking of ROS mediated plasma-induced apoptosis by D-mannitol, sodium pyruvate, carboxyl-PTIO or N-acetyl-cysteine ° Generation of different types and compositions of ROS by different plasma sources	[66]
Cervix	HeLa	° After controlled application of plasma with the precision of tens of nanometres observed killing of plasma-treated cells, neighbouring cells were not affected significantly ° Induction of morphological changes as well as indicators of apoptosis in treated cells ° Crucial role of ROS in cancer cell death induction	[67]
Cervix	HeLa	° Induction of cellular lipid membrane collapse by atmospheric-pressure plasma ° Alteration of electrical conductivity of the cells and induction of lipid oxidation by ROS	[68]
Cervix	SiHa + healthy human cervical tissue cells from cervical conus	° Immediate and persisting decrease in CC cell growth and cell viability associated with significant plasma-dependent effects on lipid structures	[69]
Endometrium	AMEC HEC50	° Reduction of cell viability and induction of cell death by PAM ° Increased autophagic cell death ° Inactivation of the mTOR pathway by PAM ° G2/M-phase arrest in all PAM concentrations ° Induction of intracellular ROS accumulation	[70]
Endometrium	HEC-1 HEC-108	° Reduction of cells containing high levels of aldehyde dehydrogenase (ALDH) - a marker of cancer-initiating cells (CICs) ° Synergistic effect of combined treatment with cisplatin, especially at lower doses ° Combination of plasma and cisplatin treatment is effective both in ALDH high and low cells	[71]
Endometrium	HEC-1 GCIY	° Reduction of cell viability ° Reduction of the number of cells with high aldehyde dehydrogenase (ALDH) production	[72]
Ovary	OVCAR-3 SKOV-3 TOV-21G TOV-112D	° Variation of anti-proliferative efficacy of CAP dependent on treatment duration as well as on the OC cell line used ° Decreased motility, invasion, and metastasis potential ° Culture medium treated with plasma before addition mediates the CAP effect on the cells, however, this effect depends on the cell medium composition	[73]
Ovary	SKOV-3 OV-90 HOSE	° Selective anticancer activity of plasma-activated Ringer’s Lactate solution (PA-RL) containing reactive oxygen and nitrogen species (RONS)	[74]
Ovary	TOV21G ES-2 SKOV3 NOS2 OHFC HPMC	° Decreased viability of CCC cell line after plasma-activated medium treatment ° Induction of morphological changes in EOC cell lines treated with PAM ° Anti-tumour effects mediated by produced ROS ° Selective anti-proliferative effect on cancer cells without causing adverse reactions in normal cells	[75]
Ovary	NOS2 NOS3 NOS2TR NOS2CR NOS3TR NOS3CR	° Decreased viability of ovarian cancer cells treated with PAM in plasma activation time-dependent manner ° Treatment with PAM decreased proliferation rate of paclitaxel and cisplatin-resistant cells derived from parental cell lines ° Addition of ROS scavenger into activated medium decreases anticancer activity, the addition of ROS scavenger inhibitor re-established anticancer activity, thus this point on the crucial role of ROS in an anti-tumour mechanism	[76]
Ovary	K2 K2R100 TOV-21G ES-2	° An anti-tumour effect of PAM on acquired chemo-resistant OC cells ° An anti-tumour effect of aqueous plasma against clear-cell carcinoma, which is natively chemo-refractory OC ° PAM has a selective cytotoxic effect on OC cells	[77]
Ovary	SKOV3 HRA	° Effective killing of ovarian cancer cells lines by the plasma, while plasma-treated fibroblast cells were not damaged ° Plasma treatment induces apoptosis ° The exposure time of treatment affects the proliferation rate	[78]
Ovary	OVCAR-3 NOS2 TOV21G ES-2	° Negative impact of cell density on PAM-induced proliferation inhibition rate ° Selective, cell line dependent sensitivity to PAM ° Dependence of PAM effect on the proportion of ROS and the cell number ° Sensitivity to PAM affected by morphological characteristics of the cells ° TGF-β induced epithelial-mesenchymal morphological transition sensitised cancer cells to PAM	[11]
Ovary	ES2 SKOV3 WI-38 HPMCs	° Inhibition of cell viability of ovarian cancer cells depends on the cell type, cell number, and plasma-activated medium (PAM) dilution ratio ° PAM mediated suppression of cell migration, invasion, and adhesion ° PAM-induced down-regulation of matrix metalloproteinase-9 (MMP-9) prevents cell plantation in co-culture with human peritoneal mesothelial cells ° Inhibition of anti-metastatic effect of PAM by the ROS scavenger	[157]
Breast	MCF-7	° CAP inhibitory effect on the cell proliferation is mediated by miR-19a-3p (miR-19a, oncomiR) ° CAP induces hypermethylation at the promoter CpG sites and subsequent downregulation of miR-19a ° CAP recovers production of ABCA1 and PTEN which are targets of miR-19a	[38]
Breast	MCF-7 MCF-7/TamR	° CAP induces restoration of sensitivity to tamoxifen (Tam) in Tam-resistant cells ° Increase of ROS levels in CAP-treated cells ° Inhibition of the proliferation and promotion of the apoptosis in MCF-7/TamR ° Oppositely altered expression of 20 genes involved in Tam resistance in TamR cells and CAP-treated TamR cells ° *MX1* and *HOXC6* mediated the restoration of sensitivity against Tam	[39]
Breast	MSC MDA-MB-231	° Synergistic inhibition of breast cancer cell growth after treatment with the combination of CAP and drug (5FU) loaded core-shell nanoparticles ° Induction of down-regulation of metastasis-related genes (*VEGF*, *MTDH*, *MMP9*, and *MMP2*) ° Facilitation of the uptake of drug-loaded nanoparticles	[40]
Breast	MCF7 MCF10A MTT	° Reduction of the viability of breast cancer cells ° Significantly lower CAP-induced damage on normal cells ° Enhanced reduction of cancer cells viability after addition of 5% oxygen to the helium plasma	[41]
Breast	metastatic BrCa cells MSC	° CAP-induced selective ablation of metastatic BrCa cells in vitro without damaging healthy MSC ° Inhibition of the migration and invasion of BrCa cells after CAP treatment ° Different BrCa cell and MSC responses under varied CAP conditions	[42]
Breast	MCF-7	° Induction of apoptosis in cultured human breast cancer cells ° Significant portion of CAP-treated cells exhibits apoptotic fragmentation, with only limited necrosis	[43]
Breast	MDA-MB-231 MCF-7 HMEC	° ROS in a liquid phase is generated via plasma irradiation of gas, producing the reactive species (electrons, ions, and radicals) and these species dissolve into the liquid phase and/or react with water ° Irradiation time, distance to the liquid surface and voltage affects OH radical generation in the extracellular culture medium	[44]
Breast	MDAMB231 MDAMB468 MCF7 MCF10A	° Induction of apoptosis, inhibition of the proliferation and migration of triple-negative breast cancers (TNBC) after PAM treatment ° Significant increase of H_2_O_2_ concentration in the media after CAP treatment ° PAM selectively inhibits the activity of JNK and NF-κB in TNBC cells	[55]
Breast	4T1	° Inhibition of cell migration after both plasma and doxorubicin treatment, assessed by wound healing assay	[56]
Breast	MCF-7 MCF-7/TxR	° Restoration of sensitivity to paclitaxel in resistant cells ° Identification of altered expression of multiple drug resistance-related genes ° *DAGLA* and *CEACAM1* were essential for the acquisition of resistance and the recovery of sensitivity	[158]

**Table 2:** A brief overview of anti-cancer effects of CAP.

Anti-Cancer Potential of CAP	Cancer Types		Study Details	Reference
Direct anti-tumour effects of CAP	Melanoma cells (Mel Im and Mel Juso)		→ calcium influx → senescence	[264]
			↑ acidification: → anti-cancer efficacy	[30]
	Melanoma cell A375 and A875		→ apoptosis (Sestrin2-mediated nitric oxide synthase signalling)	[151]
	Breast cancer cells MCF-7		Opposite regulation of ZNRD1 and its lncRNA	[265]
	Ovarian cancer cells		↓ growth and mobility	[73]
	Lung cancer cells A549		Atmospheric pressure plasma irradiation: 8-oxoguanine formation DNA strand breaks	[247]
Indirect anti-tumour effects of CAP (PAM)	Breast cancer cells SKBR3		O_3_ formation	[266]
	Triple negative breast cancer cells MDAMB231, MDAMB468 and Balb/c mice transplanted with MDAMB231 cells		→ apoptosis ↓ proliferation, migration	[55]
	Ovarian cancer cells ES2 and Balb/c mice injected with ES2		↓ migration, invasion, adhesion ↓ metastatic potential ↓ MMP9 ↓ MAPK activation ↓ phosphorylation of JNK1/2 and p38 MAPK	[157]
	Gastric cancer cells SC-2-NU, AGS, GCIY-EGFP and peritoneal dissemination mouse model using GCIY-EGFP gastric cancer cells		↓ migration, adhesion ↓ peritoneal metastatic modules	[267]
Synergy of CAP and nanotechnology	CAP + iron oxide-based magnetic NPs	Lung cancer cells A549 and Balb/c mice injected with A549 cells	↓ proliferation, viability → apoptosis ↓ xenograft tumours	[91]
	CAP + core-shell NPs	Breast cancer cells MDA-MB-231	↓ growth ↓ metastasis-related genes (VEGF. MTDH, MMP9, MMP2) → drug loaded NP uptake	[40]
	CAP + silymarin nanoemulsion	Melanoma cells	→ autophagy PI3K/mTOR and EGFR activation Modulation of transcription factors (ZKSCAN3, TFEB, FOXO1, CRTC2, and CREBBP) and autophagy-related genes (BECN-1, AMBRA-1, MAP1LC3A, and SQSTM)	[268]
	CAP + PEG-coated gold NPs	Glioblastoma T98G and lung adenocarcinoma A549 and Balb/c female nude mice injected with glioma U87MG cells	PI3K/AKT blockage EMT reversion: ↑ E-cadherin ↓ N-cadherin, Slug, Zeb-1	[269]
	CAP + gold NPs	Colon cancer cells HCT-116	↓ cell deaths	[270]
	CAP + platinum NPs	Human lymphoma U937 cells	Attenuated CAP-induced ROS-mediated apoptosis	[271]
	CAP + gold NPs	Glioblastoma multiforme U373MG cells	→ clathrin-dependent endocytosis to repair oxidised membrane → uptake of nanomaterial	[48]
	CAP + gold NPs	Glioblastoma multiforme U373MG cells	Activation of NPs toxicity ↑ endocytosis ↑ trafficking to lysosomes	[272]
	CAP + paclitaxel-loaded core-shell magnetic NPs	Non-small cell lung cancer cells A549	↓ growth	[273]

Explanatory notes: ↑ increase; → promotion, induction; ↓ decrease; + plus. Abbreviations: CAP, cold atmosphere plasma; EMT, epithelial-mesenchymal transition; lncRNA, long non-coding RNA; NPs, nanoparticles; PAM, plasma-activated medium.
